# Jet cross sections at the LHC and the quest for higher precision

**DOI:** 10.1140/epjc/s10052-019-7574-x

**Published:** 2020-02-05

**Authors:** Johannes Bellm, Andy Buckley, Xuan Chen, Aude Gehrmann-De Ridder, Thomas Gehrmann, Nigel Glover, Stefan Höche, Alexander Huss, Joey Huston, Silvan Kuttimalai, Joao Pires, Simon Plätzer, Emanuele Re

**Affiliations:** 1 Department of Astronomy and Theoretical Physics, Lund University, 223 62 Lund, Sweden; 2 School of Physics and Astronomy, University of Glasgow, Glasgow, G12 8QQ UK; 3 Institut für Theoretische Physik, Universität Zürich, 8057 Zürich, Switzerland; 4 Institute for Theoretical Physics, ETH, 8093 Zürich, Switzerland; 5 Institute for Particle Physics Phenomenology, Durham University, Durham, DH1 3LE UK; 6 SLAC National Accelerator Laboratory, Menlo Park, CA 94025 USA; 7 Fermi National Accelerator Laboratory, Batavia, IL 60510-0500 USA; 8 Theoretical Physics Department, CERN, 1211 Geneva 23, Switzerland; 9 Department of Physics and Astronomy, Michigan State University, East Lansing, MI 48824 USA; 10 CFTP, Instituto Superior Técnico, Universidade de Lisboa, 1049-001 Lisboa, Portugal; 11 Fakultät Physik, University of Vienna, 1010 Vienna, Austria; 12 Laboratoire d’Annecy-le-Vieux de Physique Théorique, Université Grenoble Alpes, Université Savoie Mont Blanc, CNRS, 74940 Annecy, France

## Abstract

We perform a phenomenological study of *Z* plus jet, Higgs plus jet and di-jet production at the Large Hadron Collider. We investigate in particular the dependence of the leading jet cross section on the jet radius as a function of the jet transverse momentum. Theoretical predictions are obtained using perturbative QCD calculations at the next-to and next-to-next-to-leading order, using a range of renormalization and factorization scales. The fixed order predictions are compared to results obtained from matching next-to-leading order calculations to parton showers. A study of the scale dependence as a function of the jet radius is used to provide a better estimate of the scale uncertainty for small jet sizes. The non-perturbative corrections as a function of jet radius are estimated from different generators.

## Introduction

The production of a single object like a *Z* or Higgs boson, or a jet, at high transverse momentum has been studied intensely in hadron collider environments, both theoretically and experimentally. These processes are used for measuring standard-model parameters, to constrain parton distribution functions (PDFs), and to understand backgrounds to new physics searches. They probe the structure of the QCD interactions in great detail. On the one hand, the large scales associated with the production of a high-$p_T$ object make QCD perturbation theory a prime analysis tool. For $H/Z+\ge 1$ jet production, the large boson mass also provides a large scale to further stabilize the QCD prediction. On the other hand, the exclusive nature of the reactions may induce logarithmically enhanced higher-order corrections, which must be resummed to all orders. Both aspects must be incorporated into simulations used for experimental and phenomenological analyses to provide accurate predictions.

In the past few years, the state of the art in QCD perturbation theory has advanced considerably. Next-to-next-to-leading order QCD predictions are now available for *Z*-boson plus jet [1–5], Higgs-boson plus jet [6–10], and for inclusive jet and di-jet production [11–15]. Next-to-leading order accurate results have been available for some time [16–18]. They can now be computed in an automatic fashion using general-purpose event generators [19–27] and the matching to parton showers can be carried out with a number of different approaches [28, 29]. Analytic results for jet radius [30] and combined jet radius and threshold resummation are available as well [31, 32].

A contribution to the Les Houches 2017 workshop compared predictions for H+j production at LO, NLO and NNLO to those from parton-shower matched NLO calculations using different event generators, for a variety of jet radii [33]. The goal for this comparison was multi-fold: using identical boundary conditions, to check the consistency of the matched predictions, and to demonstrate that the matched results revert to their underlying fixed order predictions in kinematic regions where Sudakov resummation can be neglected. This was inspired by previously observed large discrepancies between various parton-shower simulations and NLO-matched predictions [34]. Good agreement was observed between different parton showers, and between the matched predictions and the fixed order results. The best agreement of the jet shape was obtained in the comparison between NLO+PS matched and fixed-order NNLO predictions, which is expected given the more complete description of the jet shape upon including double-real radiative corrections at NNLO.

In this study, we follow up with further comprehensive comparisons for Higgs+jet, and for two additional processes, *Z*+jet and dijet, concentrating on inclusive observables, such as the lead jet transverse momentum distribution. Contrary to popular opinion, the agreement among the various NLO+PS matched predictions (including POWHEG for dijet production) for these observables is good, as is the underlying agreement with the relevant fixed order calculation, especially at NNLO, if each prediction is used properly, and with identical parameters. One of the powerful and indeed unique aspects of this study is the comparison of jet cross sections for a wide variety of jet radii (beyond what is commonly used by the ATLAS and CMS experiments). This allows for a better fundamental understanding of the underlying physics, both perturbative and non-perturbative.

In addition, we examine the scale uncertainties of the three processes, at LO, NLO and NNLO, as a function of jet radius, and comment on the implication of our results on the determination of *reasonable* scale uncertainties.

The paper is organized as follows. In Sect. 2 we detail the setup of the generators used in this study. In Sect. 3 we discuss the the shape of jets for the three processes. Section 4 focuses on the transverse momentum dependence of the cross-sections and the influence of higher order corrections at fixed order. In Sect. 5 we describe and compare results from the fixed order expansion to parton level Monte Carlo simulations. In Sect. 6 we consider cross section uncertainties that arise due to the jet definition. Before we give concluding remarks and an outlook in Sect. 8 we examine the possible influence of hadronization and multiple parton interactions on the measurement of cross-sections in Sect. 7.

## Setup

We investigate Higgs+jet, *Z*+jet and inclusive jet production, taking advantage of the NNLO calculations available for all three processes. The latter two reactions are important for global PDF fits, where only fixed order predictions (along with the relevant non-perturbative corrections) have been used so far, and thus it is important to understand the possible impact of resummation effects.

The analyses use the anti-$k_T$ jet algorithm [35], with varying jet size as described below, with a jet transverse momentum threshold of 30 GeV, along with a cut on the jet rapidity of 4.5. To avoid generation cut effects, the comparisons are performed above a jet transverse momentum of 50 GeV. Further, any cross-section that is sensitive to the colourless system is to be taken above a transverse momentum of 90 GeV to ensure the possibility of having three well-separated partons (at NNLO) recoiling and resulting in generation cut migration.

As a further test of the impact of parton showers versus fixed order, the jet size was varied across the values 0.3, 0.4, 0.5, 0.6, 0.7, and 1.0, using the anti-$k_T$ jet algorithm. Indirectly, this tests how well the one (two) extra parton(s) at NLO (NNLO) reproduce resummation effects. This is of particular interest as the Higgs (*Z*) boson + jets measurements that have been performed at the LHC in Run 2 have typically used a jet size of 0.4, which is only slightly above the region where small *R* effects become important.^1^ Taking the small *R* effects into proper account would require resummation, as discussed in [30–32]. The NLO+PS predictions provide this resummation by means of the parton showers.

In this study, predictions from NLO+PS programs were carried out at the parton shower level to make them comparable to the fixed order calculations.^2^ To the degree to which it was possible, the fiducial setups have been constrained to be the same for all calculations. We used the PDF4LHCNNLO_30 PDFs [36], with its central value of $\alpha _s(m_Z)$ of 0.118. We do not address PDF uncertainties. The renormalization and factorization scales used to compute the fixed-order perturbative results have been chosen as similar as possible, providing a greater level of control than was available in a similar study during the 2015 Les Houches workshop [37]. More details will be provided in the sub-sections. We use the Rivet framework [38] to analyze events. A CMS routine from the 13 TeV inclusive jet analysis [39] was modified to add the different *R* values, as well as additional observables.^3^

### NNLOJET

The NNLO corrections to $pp \rightarrow X+j$ receive contributions from three types of parton-level subprocesses: the *X*+5 parton tree-level (double-real correction), the *X*+4 parton process at one-loop (real–virtual correction), and the two-loop *X*+3 parton result (double-virtual correction). The double-real, real-virtual and double virtual corrections to $pp \rightarrow H+j$ production were computed in [40–45] and [46], respectively. The double-real, real-virtual and double virtual corrections to $pp \rightarrow Z+j$ production were computed in [47–54] and [55–58], respectively. The double-real, real-virtual and double virtual corrections to inclusive jet and di-jet production were computed in [59–62] and [63–69], respectively.

Each of the above components of the NNLO calculations is separately infrared (IR) divergent, and the divergences cancel upon integration over the unresolved phase space by virtue of the Kinoshita–Lee–Nauenberg theorem. In order to compute a fully differential prediction using Monte-Carlo integration techniques, a procedure for the subtraction of IR singularities is required to make this cancellation manifest, and to construct a locally finite integrand. To this end, we employ the antenna subtraction formalism [70–78], which is implemented in the NNLOJET framework.

For the central predictions of our current study, we use the following dynamical scale for *H*+jet and *Z*+jet production processes,

1 $$\begin{aligned} \mu _0 = \frac{H_{T}}{2} = \frac{1}{2}\bigg (\sqrt{m_X^2+p_{T,X}^2} +\sum _\mathrm{partons} p_{T,j}\bigg ). \end{aligned}$$

The LO and NLO differential cross sections using this dynamical scale choice were validated against Sherpa and Herwig7. The renormalisation ($\mu _R$) and factorisation ($\mu _F$) scales are varied independently around $\mu _0$ by factors of $\tfrac{1}{2}$ and 2 to estimate the size of missing higher-order contributions. Here, the two extreme variations are excluded such that we arrive at the custom 7-point scale variation [79]:

2 $$\begin{aligned}&(\mu _R,\mu _F) \nonumber \\&\quad = \bigl \{ (1,1), \; (2,2), (\tfrac{1}{2},\tfrac{1}{2}), \; (\tfrac{1}{2},1), \; (1,\tfrac{1}{2}), \; (2,1), \; (1,2) \bigr \} \nonumber \\&\quad \qquad \times \mu _0 . \end{aligned}$$

The inclusive jet production process has been studied at NNLO in Ref. [80], using a standard scale choice of $\mu _0=p_T^\mathrm{jet}$ where this quantity refers to the transverse momentum of each individual jet. Thus, for each jet in a NNLO event, there is a corresponding entry in the plot with the matrix element weight evaluated at the jet $p_T$ as the scale. This is the very definition of an inclusive cross section. An alternative choice is to use as a scale the transverse momentum of the highest $p_{T}$ jet in the event ($p_{T}^\mathrm{jetmax}$) [13]. The use of these two scales creates a sizeable difference at NNLO at low transverse momentum [15], which is larger than the nominal scale uncertainty around either scale. This effect was examined in detail in Ref. [15], leading to the observation that large-scale cancellations between different kinematical configurations in the second jet contribution are aggravated for certain scales. An event based scale ($H_{T}$ see Eq. (1)) built from the scalar sum of transverse momenta of all partons in the event was found to be stable, leading to an improved perturbative convergence on the transverse momentum distributions, with overlapping scale uncertainty bands between NLO and NNLO. In this study this is the default scale choice to perform a standard comparison with the ME+PS predictions, unless otherwise stated in the text.

In order to obtain the results for the various jet sizes in Higgs+jet and *Z*+jet ($R=0.3$, 0.4, 0.5, 0.6, 0.7, and 1.0) required in this study, we have exploited the fact that the Born-level kinematics for all processes is insensitive to *R*. As a result, the difference between two cone sizes can be obtained from a calculation of the *H*+2 jet and *Z*+2 jet process at NLO accuracy:

3 $$\begin{aligned}&\sigma ^\mathrm{NNLO}_{H(Z)+j}(R) -\sigma ^\mathrm{NNLO}_{H(Z)+j}(R') \nonumber \\&\quad = \int \bigl [\mathrm {d}\sigma ^\mathrm{NLO}_{H(Z)+2j}(R) -\mathrm {d}\sigma ^\mathrm{NLO}_{H(Z)+2j}(R') \bigr ]_{N_\mathrm{jets} \ge 1} . \end{aligned}$$

Note that the difference has to be taken at the level of the integrand, since one term acts as a local counter-term of the other in all IR-divergent limits where a jet becomes unresolved and the *H*+2 jet / *Z*+2 jet configuration degenerates to *H*+jet / *Z*+jet. Using Eq. (3), predictions for different *R* values can be obtained from a single NNLO computation.

### Setup for Sherpa

We use a pre-release version of the Sherpa Monte Carlo event generator [81, 82], based on version Sherpa-2.2.4. The NLO matching is performed in the S-MC@NLO approach [83, 84]. We use a modified version of a parton shower algorithm [85], which is based on Catani-Seymour dipole subtraction [86, 87]. We use a running coupling consistent with the PDF, and employ the CMW scheme to include the two-loop cusp anomalous dimension in the parton-shower simulation [88]. To make the result comparable to the fixed-order predictions we set the renormalization and factorization scales as Eq. (1) to

4 $$\begin{aligned} \mu _0= & {} \frac{H_T}{2} = \frac{1}{2}\bigg (\sqrt{m_X^2+p_{T,X}^2} +\sum _\mathrm{partons} p_T\bigg ) \nonumber \\&\quad \text {for Higgs/Z+jet production}, \end{aligned}$$

5 $$\begin{aligned} \mu _0= & {} {H}_T = \sum _\mathrm{partons} p_T\;\qquad \text {for inclusive jet production}, \end{aligned}$$

and we set the resummation scale to $(p_{T,H/Z}+\sum _\mathrm{partons} p_{T})/2$ for Higgs/Z+jet production and to $\sum _\mathrm{partons} p_{T}/2$ for inclusive jet production.

### Setup for Herwig

We used Herwig 7 [25, 89–91] based on version 7.1.4 and ThePEG version 2.1.4 with minor changes to standard Herwig 7 scale settings to match Eq. (4). The NLO matching was performed with matrix elements from OpenLoops [22] and MadGraph [92] interfaced using the BLHA2 standard [93]. For parton distributions the PDF interface from LHAPDF6 [94] was used. In the results we show matched $\mathrm {NLO} \oplus \mathrm {PS}$ predictions with the $\tilde{Q}$-shower [95]; using lower statistics it was confirmed that merging according to [96, 97] and matching to the Herwig 7 dipole shower [98] display similar behaviour. For parton level comparisions, hadronization and MPI models were switched off and the $\alpha _S$ of the hard process was synchronized with the PDF set. We include the effects of the CMW scheme [88] by an enhanced shower $\alpha _S=0.124$. The scale used for the core process in the matching is defined as in Eq. (4), and the resummation scale was set to the transverse momentum of the hardest jet.

### Setup for POWHEG BOX

Inclusive jet production was simulated using POWHEG BOX (v2) [29, 99, 100], using the implementation described in Ref. [101]. The hard matrix elements entering the $\bar{B}$ function have been evaluated using the scale choice in Eq. (5). In order to match the setup of Sherpa and Herwig7, we used the options btlscalereal 1 and btlscalect 1, thereby computing the real matrix elements using values of $\mu _R$ and $\mu _F$ obtained using the corresponding phase space kinematics, rather than the underlying Born one. The running of the strong coupling is consistent with the PDF choice, and the CMW scheme is used in the POWHEG Sudakov form factor. The partonic events were then showered using Pythia8 (version 8.230) [102], using the default tune and hence the default PDF choice for the showering stage. For this study, vetoing in parton showering has been achieved using the settings “SpaceShower:pTmaxMatch = 1” and “TimeShower:pTmaxMatch = 1”.

**Fig. 1:**
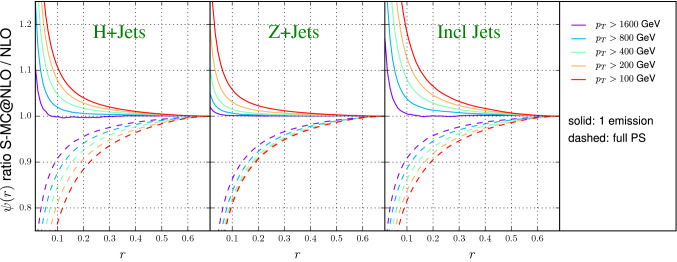
Jet shapes for Higgs and *Z* plus jets and inclusive jets

## Jet shapes

In Fig. 1, we investigate the difference between the fixed-order NLO and NLO matched predictions for integrated jet shapes for Higgs+jets, *Z*+jets and dijet production. The integrated jet shape is defined as

6 $$\begin{aligned} \Psi (r) = \frac{1}{N^\mathrm{jet}}\sum \limits _{\mathrm{jets}} \frac{p_\mathrm{T}(0,r)}{p_\mathrm{T}(0,R)}, \end{aligned}$$

with *r* being the radius of a cone which is concentric to the jet axis and $p_\mathrm{T}(r_1,r_2)$ being the magnitude of the scalar sum of transverse momenta in the annulus between radius $r_1$ and $r_2$. We also compare to a parton-shower matched prediction, where the number of final-state partons generated in the simulation is limited to at most two. This simulation presents the closest possible approximation to the fixed-order NLO result that we are able to generate using the matching algorithms. It reflects the kinematical restrictions of the NLO calculation (i.e. that only up to one additional final-state parton can be present), but it also includes additional approximate higher-order virtual corrections by means of Sudakov factors. Nevertheless, we observe that the full NLO result and the truncated matched result approach each other well within the jet cone, and the convergence is naturally faster for larger jet transverse momenta. Note that the truncated matched result approaches the full NLO result from above, which indicates that the NLO calculation predicts less radiation close to the center of the jet. This is explained by the following effect: The real-emission contribution in the fixed-order calculation diverges as $r\rightarrow 0$, while it smoothly approaches zero in the parton-shower matched result, due to Sudakov suppression. If the parton-shower approximation to the real-emission cross section is good, this implies that at any given value of *r*, the fixed-order prediction for the differential jet shape will be larger than the parton-shower result, and conversely, that the fixed-order prediction for the integrated jet shape will be smaller than the parton-shower result. This effect is somewhat reduced by the different scale choice in the two calculations, but it can still be observed in Fig. 1.

In the following we see many examples where there is a differences compared to the good agreement between the truncated matched prediction and the fixed-order calculation. It must be suggested that higher-multiplicity final states are responsible for these differences. The discrepancies at small and large *R* should therefore be reduced for higher-order perturbative calculations, especially at NNLO. The jet shape for the *Z*+jets process is noticeably narrower. We attribute this to the lead jet accompanying the *Z*-boson being predominantly a quark jet, which is more collimated than a gluon jet due to its reduced color charge.

## K-Factors and R-dependence at fixed order

**Fig. 2:**
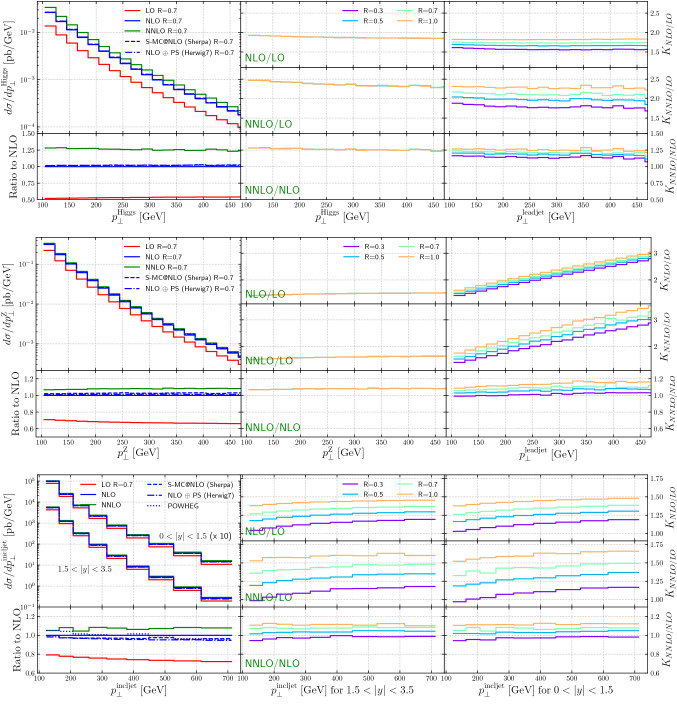
*K*-factors for Higgs plus jets (top), *Z* plus jets (middle) and inclusive jet production (bottom)

Figure 2 top (middle) shows (left) the transverse momentum spectrum of the Higgs boson (*Z*-boson) as predicted by the fixed-order LO, NLO and NNLO calculation, as well as the results from an NLO matched computation using the Sherpa event generator and the NLO-matched Herwig result. The NLO, Sherpa and Herwig results are all in very good agreement with each other over the entire range of the plot ($\ge$ 100 GeV). For these given processes and observables the correction at the NNLO level results in an increased (differential) cross-section.

Figure 2 top (middle) shows (center) the *K*-factors (NLO/LO, NNLO/LO, NNLO/NLO), from NNLOJET as a function of the Higgs boson (*Z*-boson) $p_T$, for different jet radii; as expected there is no jet size dependence for this variable in the plotted region. Also shown (right) are the local *K*-factors as a function of the lead jet transverse momentum for the two processes, for various jet sizes. The *K*-factors for $H+\ge 1$ production are relatively flat as a function of jet $p_T$. The *K*-factors (NLO/LO and NNLO/LO) for $Z+\ge 1$ production grow rapidly with jet $p_T$, due to the increasing dominance of dijet production, followed by a *Z*-boson emission. The *K*-factors (NNLO/NLO) are relatively flat, indicating that there are no substantial new subprocesses being added at NNLO.

Figure 2 (bottom left) shows the inclusive jet transverse momentum spectrum as predicted by the fixed-order LO, NLO and NNLO calculations, for an *R*-value of 0.7, as well as the results from an NLO matched computation using the Sherpa event generator and the NLO-matched Herwig result. In addition, a prediction from Powheg is included as well. The NLO, Sherpa, Herwig and Powheg results are all in very good agreement with each other over the range of the plot ($$\ge $$ 100 GeV), i.e. there is no significant $$ parton$$ $$ shower$$ $$ systematic$$ and the predictions with parton showers reflect the underlying fixed-order NLO results. The NNLO normalizations are larger due to the higher order effects included in these calculations. *K*-factors (NLO/LO, NNLO/LO, NNLO/NLO, from NNLOJET are shown as a function of jet size, and as a function of the inclusive jet $$p_T$$, for two different rapidity intervals. Again, the *K*-factors grow with increasing jet size, and also have a slight slope (NLO/LO, NNLO/LO) as a function of the jet transverse momentum.

**Fig. 3:**
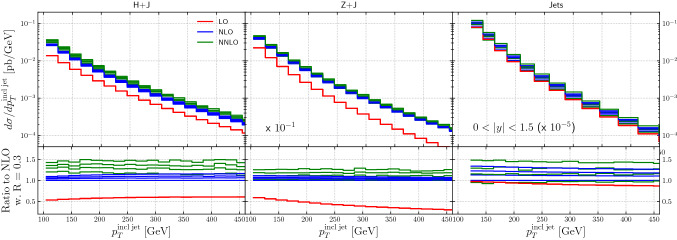
The cross sections for $H +\ge 1$ jet, $Z +\ge 1$ jet, and dijet production from NNLOJET, as a function of the inclusive jet $p_T$ at LO, NLO and NNLO. To illustrate the spread induced on the cross section, representative values of $R\in [0.3,0.5,0.7,1.0]$ are shown

The cross sections for $H +\ge 1$ jet, $Z +\ge 1$ jet, and dijet production from NNLOJET are shown in Fig. 3, as a function of the inclusive jet $p_T$ at LO, NLO and NNLO. The figure shows representative values for $R\in [0.3,0.5,0.7,1.0]$ to illustrate the spread induced on the cross section. It is interesting to note that for $H +\ge 1$ jet production, the *R*-dependence is larger at NNLO than at NLO. The *R*-dependence for $Z +\ge 1$ jet production is – compared to Higgs production – relatively small both at NLO and NNLO. For dijet production, the *R*-dependence is relatively large at both NLO and NNLO. The larger *R*-dependence for $H +\ge 1$ jet production at NNLO than at NLO can be traced back to the large radiative corrections to the signal at NLO. A significant part of the large higher-order corrections in inclusive *Z*- and Higgs-boson production originates in the well-known ratio of the Sudakov form factor between the timelike and the spacelike region [103]. This ratio, given by $\left| \Gamma _a(Q^2)/\Gamma _a(-Q^2)\right| ^2=1+\alpha _s(Q^2)/(2\pi )\,C_a\pi ^2+\mathcal {O}(\alpha _s^2)$ is enhanced by the color charges of the partons annihilating into the electroweak boson and is therefore larger in Higgs- than in *Z*-boson production. While the analysis is more complicated in processes with an additional final-state jet, universal terms of the same form are present there, which might explain the larger NNLO / NLO *K*-factors in the case of Higgs-boson plus jet production [104].

**Fig. 4:**
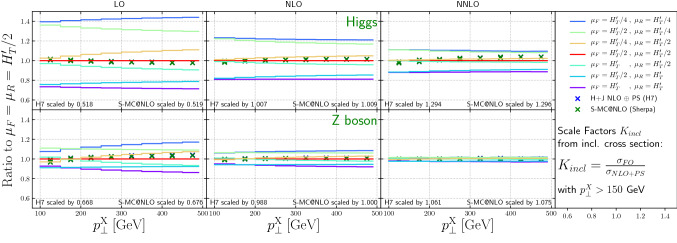
The scale variations at LO, NLO, and NNLO from NNLOJET for Higgs and *Z* boson production, as a function of the boson transverse momentum, are shown. For comparison, the nominal NLO NLO + PS predictions are also shown. The generator predictions are scaled with the inclusive $K_{incl}$ factor with Higgs(Z) $p_\perp > 150$ GeV, see Fig. 2

## Results: fixed order vs. parton level Monte Carlo

Figure 4 shows the scale dependence of the differential cross section as a function of the Higgs or *Z*-boson transverse momentum. The NLO-matched parton shower predictions have been scaled by *K*-factors derived from the constraint that the inclusive cross section for heavy boson transverse momenta above $p_\perp > 150$ GeV match the fixed-order result. The reduction of the scale dependence in the transition from LO to NNLO is striking. It is also encouraging that the scaled NLO-matched parton shower calculations agree very well with the fixed-order results over the entire range in transverse momentum. This implies that the Monte-Carlo generators can be utilized to reliably predict the heavy boson transverse momentum spectra in boosted Higgs and *Z*-boson analyses.

If the renormalization and factorization scales are defined using partonic variables, we expect a very mild dependence of the Higgs/*Z* transverse momentum spectrum on both the jet $p_T$ cut and the jet radius in the plotted region. At leading order QCD, this transverse momentum is compensated by a single hard jet. The collinear evolution of the jet is governed by the DGLAP equations, which prefer highly asymmetric branchings of the jet into softer sub-jets. Only if this evolution reaches the extremely unlikely final-state configuration with all jets below the $p_T$ threshold or outside the rapidity region covered by the detector, the event can be lost and the cross section can be changed. Due to the restricted final-state multiplicity, the probability for this is even more reduced at fixed order. In fact, without any jet rapidity cuts, the cross section could not be modified up to N${}^5$LO, where the opportunity arises for the first time to have all partons forming individual jets at $p_T=150\;\mathrm{GeV}/6<30\;\mathrm{GeV}$, albeit in a very small phase space.

**Fig. 5:**
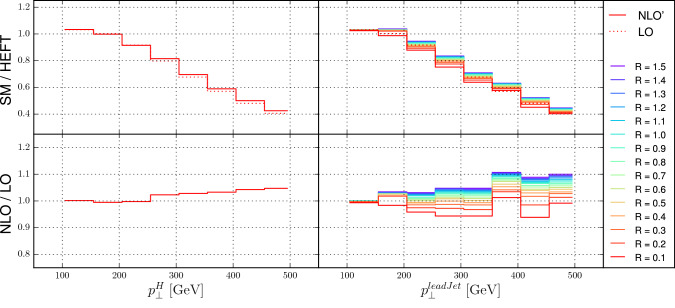
The ratio between results computed in HEFT and the full Standard Model for the transverse momentum spectrum of the Higgs boson (left) and the leading jet (right) in Higgs plus jet events. Results labeled NLO’ are derived using the approximation of [105]

In the context of Higgs boson measurements at high transverse momentum, the difference between predictions computed in the Higgs effective theory (HEFT) and the full Standard Model, (including dependence on the top-quark mass), becomes important. The full Standard Model features a significantly steeper transverse momentum spectrum as well as different scale uncertainties. A complete calculation at NNLO precision in the full Standard Model is currenly out of reach. One can, however, assume that top-quark mass effects factorize from higher-order QCD corrections, such that they can be treated independently. In Fig. 5 we test this hypothesis, both for the Higgs boson and leading jet transverse momentum spectrum. The results labeled NLO’ are derived using an approximate virtual correction [105]. This approximation is motivated by the good agreement between such approximations and the full NLO result observed in [106]. We note that the ratio between the full SM and the HEFT result behaves very similar at LO and at NLO’, both as a function of the Higgs and the leading jet transverse momentum. Note that it has a jet radius dependence as a function of the leading jet transverse momentum. This originates in the different $p_T$ dependence of the cross section in the real-emission and Born kinematics at NLO.

**Fig. 6:**
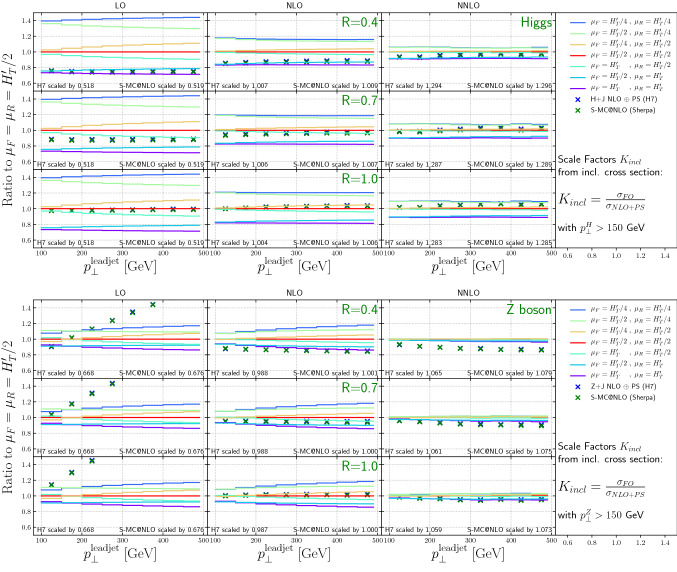
The scale variations at LO, NLO, and NNLO from NNLOJET for 3 jet sizes, as a function of leading jet transverse momentum, are shown. For comparison, the nominal NLO NLO+PS predictions are also shown. The generator predictions are scaled with the inclusive $K_{incl}$ factor with Higgs(Z) $p_\perp > 150$ GeV, see Fig. 2

Figure 6 top (bottom) panel shows the cross section scale variations at LO, NLO and NNLO for $H(Z) +\ge 1$ jet production, as a function of the leading jet transverse momentum, for various jet sizes. Normalized to the respective central scale choice we add the 7 point variation defined in Eq. 2. In both cases the symmetric variation in renormalisation scale and factorisation scale generates the largest variation. As expected, the uncertainties on the cross sections decrease from LO to NLO to NNLO.

The scale uncertainties for $H +\ge 1$ jet production are relatively constant as a function of the lead jet transverse momentum. For $Z +\ge 1$ jet production, the NLO scale uncertainties increase as the lead jet transverse momentum increases. This can be understood as the effect of new kinematic channels, which correspond to the radiation of a soft *Z* boson off a hard di-jet event generating back-to-back topologies. Such configurations arise as part of the real emission contribution in the NLO result, and they become more important as the lead jet transverse momentum increases.

Also shown for comparison are the predictions from the two NLO+PS calculations. For the sake of shape comparison, we scale these predictions with inclusive *K*-factors received by numerically integrating the differential Higgs(Z) $p_T$ distribution above 150 GeV. This has two reasons: First, as described earlier the generation cut on the jet requires high enough boson $p_T$ cuts to mitigate simulation setup effects. Second, in the parton shower simulation, we use the jet transverse momentum to define the shower starting condition. If the Higgs(Z) mass is of similar size this choice of scale would have an increased ambiguity, which could, however, be eliminated by performing a multi-jet merged computation. For $H +\ge 1$ jet production, the two NLO+PS predictions agree at a 2% level with each other and tend to be at the lower end of the scale uncertainty bands for *R* = 0.4, at center of the scale uncertainty bands for *R* = 0.7 and slightly above the center of the scale uncertainty bands for *R* = 1.0. For $Z +\ge 1$ jet production, the NLO+PS predictions again are in agreement with each other, but rapidly increase over the LO results as the lead jet $p_T$ increases (again due to the impact of the dijet contribution, arising only at NLO or above). At NLO, a similar behavior with respect to the NLO scale uncertainty band is observed as was seen for $H +\ge 1$ jet. At NNLO, the NLO+PS predictions are close to the scale uncertainty bands, which are at a $\pm 5\%$ level, especially for *R* = 0.4. This will be discussed further in the context of Fig. 8 and in Sect. 6.

**Fig. 7:**
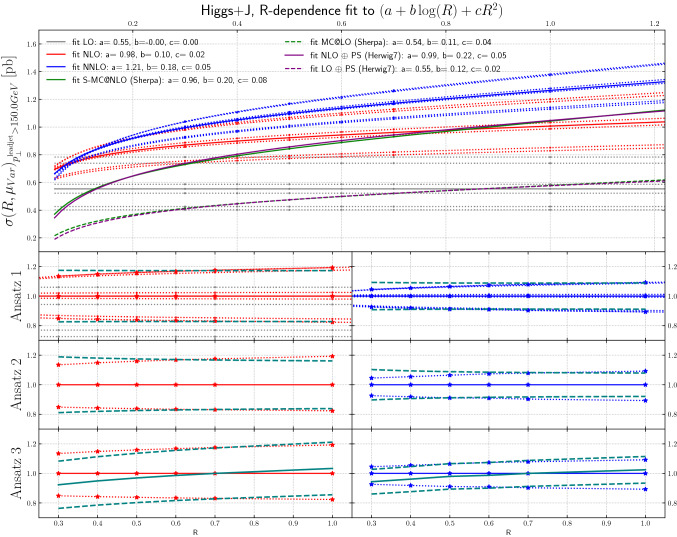
The *R*-dependence of the cross sections at NLO, NNLO and NLO + PS are shown, for particular scale values, as a function of the jet radius, for $H +\ge 1$ jet production, for leading jet transverse momenta above 150 GeV

**Fig. 8:**
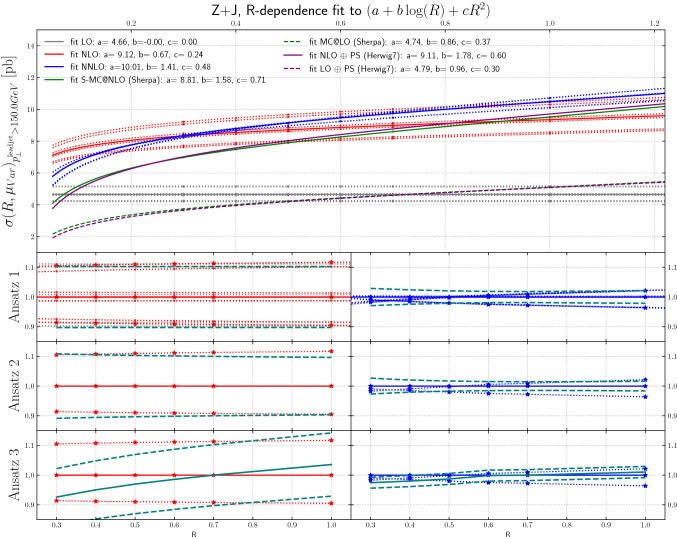
The *R*-dependence of the cross sections at NLO, NNLO and NLO + PS are shown, for particular scale values, as a function of the jet radius,for $Z +\ge 1$ jet production, for leading jet transverse momenta above 150 GeV

Figure 7 shows the leading jet $p_T$ cross sections for $H +\ge 1$ jet production for the different scale choices, at LO, NLO and NNLO, as a function of the jet size *R*.^4^ In this case, a minimum transverse momentum requirement of 150 GeV has been placed on the leading jet. We assume this scale to be large enough to replace $M_H$ as the largest scale in the process, see discussion of Fig. 6. The dots for each scale choice have been fit to a functional form motivated by the expected behavior for jet cross sections. We assume the leading functional form [110]:

7 $$\begin{aligned} f(R)=a+b\log (R)+c R^2 \end{aligned}$$

which is motivated by the logarithmic behaviour scaling of the cross section with the jet size *R* and an area-dependent contribution from initial-state radiation. The lines in Fig. 7 are then interpolations with Eq. (7) and the fitted values. Again, the scale variation band is given by the upper and lower curves at each order. It is notable that the scale uncertainty bands shrink as the jet size decreases, as mentioned earlier. For very low values of *R*, this improvement in the uncertainty can be regarded at least partially due to accidental cancellations that stem from the restrictions in phase space. It can also be observed that for each particular scale, the slope is greater at NNLO than at NLO. The NLO+PS predictions are also plotted in the figure, and can be observed to have a greater slope than even the NNLO predictions. This can be seen as an effect of either including (at large *R*) or not excluding (at small *R*) additional semi-hard real emissions, which have a leading-order scale dependence and therefore induce a large change in the cross section. The ratio panels of Figs. 7, 8 and 11 will be discussed in Sect. 6 in the context of improved scale uncertainties.

Figure 8 shows the leading jet $p_T$ cross sections for $Z +\ge 1$ jet production for the different scale choices, at LO, NLO and NNLO, as a function of the jet size *R*, and again with a minimum transverse momentum requirement of 150 GeV placed on the leading jet. The behavior at NLO is similar to what was observed for Higgs + jet. As for Higgs + jet, there is a large decrease of the scale uncertainty at NNLO at all *R* values. In fact, the scale uncertainty decreases to zero at *R* = 0.3, emphasizing the accidental cancellations noted for Higgs+jet. This may indicate that the especially small scale uncertainties for *R*=0.4, as observed for example in Fig. 6, may be underestimated.

**Fig. 9:**
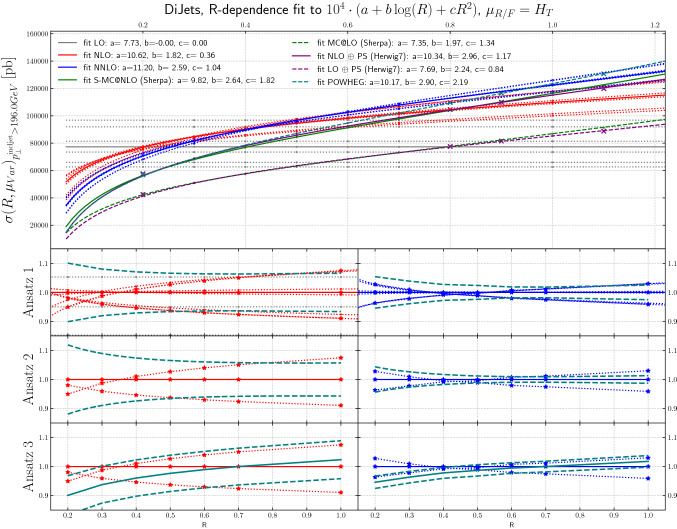
The *R*-dependence of the cross sections for inclusive jet production at LO, NLO, NNLO and NLO+PS are shown, for scale variations around a central scale of $H_T$, as a function of jet radius, for dijet production, for leading jet transverse momenta above 196 GeV

**Fig. 10:**
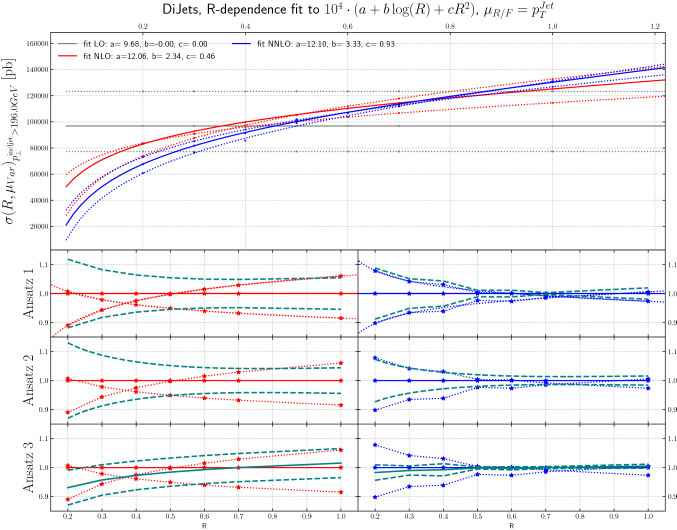
The *R*-dependence of the cross sections for inclusive jet production at LO, NLO, NNLO and NLO + PS are shown, for scale variations around a central scale of $p_T^\mathrm{jet}$, as a function of jet radius, for dijet production, for leading jet transverse momenta above 196 GeV

**Fig. 11:**
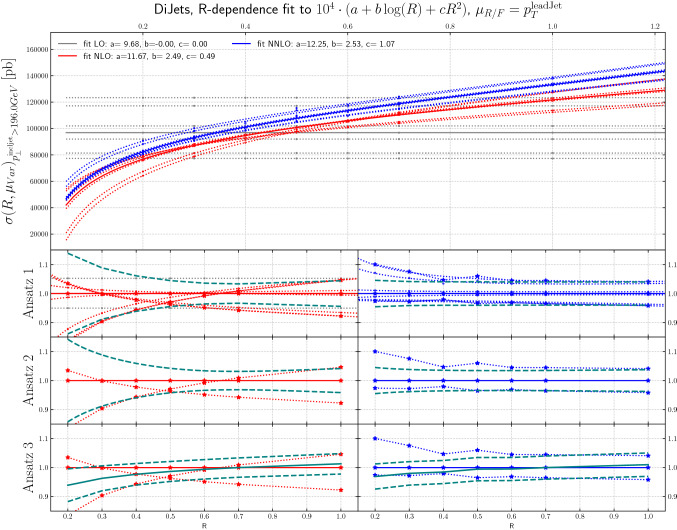
The *R*-dependence of the cross sections for inclusive jet production at LO, NLO, NNLO and NLO+PS are shown, for scale variations around a central scale of $\mu _{R/F}=p^{\mathrm{lead\;jet}}_T$, as a function of jet radius, for dijet production, for leading jet transverse momenta above 196 GeV

Figures 9, 10 and 11 show the inclusive jet cross section from dijet production, again at LO, NLO and NNLO, as a function of *R*, using scale variations around a central scales of $H_T$, $p_T^\mathrm{jet}$ and $\mu _{R/F}=p^{\mathrm{lead\;jet}}_T$, respectively. Here, the behavior is in some sense more extreme in that e.g. for the scale choice $H_T$ the jet *R* value for essentially zero scale uncertainty is at *R* = 0.4 which is one of the jet sizes that is commonly used at the LHC.^5^

**Fig. 12:**
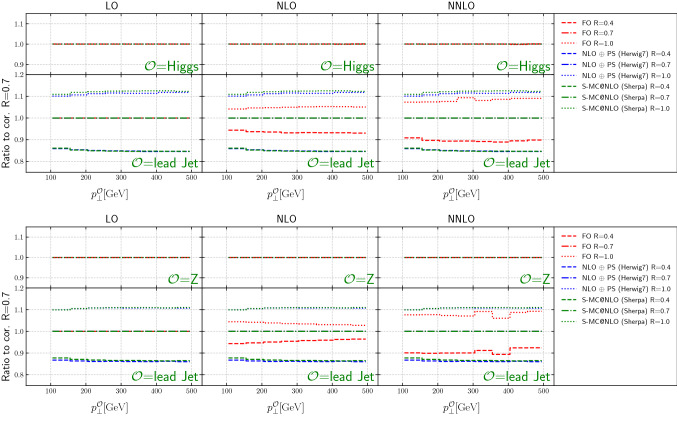
The ratio of each cross section (either Higgs(*Z*) $p_T$ or lead jet $p_T$) for specific jet sizes, scaled to the cross section for each prediction for a jet size of $R=0.7$

Figure 12 shows the cross sections for the Higgs(Z) transverse momentum and leading jet transverse momentum for several different jet sizes, at LO, NLO and NNLO (from NNLOJET) and from the two NLO+PS predictions. All cross sections have been scaled to their respective value for the reference jet size of $R=0.7$. Near this value we observe the best agreement between fixed-order and NLO matched results, save for an overall normalization which can be extracted from the Higgs(*Z*) transverse momentum spectrum, cf. Fig. 2.

The absolute value of the difference between the fixed-order and the NLO matched predictions away from $R=0.7$ increases roughly proportional to $\log (R/0.7)$ (cf. Fig. 14), which is expected due to the higher-multiplicity emissions included in the PS simulations. Depending on kinematics they either enhance (at $R>0.7$) or reduce (at $R<0.7$) the cross section.

The differences between the NLO+PS predictions and those from NNLOJET decrease as the order is raised from NLO to NNLO for both Higgs and *Z*-boson production. The difference for Higgs boson production is of the order of 5–10% for $R=0.4$ at NLO and of the order of less than 5% at NNLO, relatively flat with $p_T$. For *Z*-boson production, the differences between the NLO + PS predictions and those from NNLOJET at NLO slightly increase with increasing $p_T$, and are relatively flat and at the 3–5% level at NNLO.

**Fig. 13:**
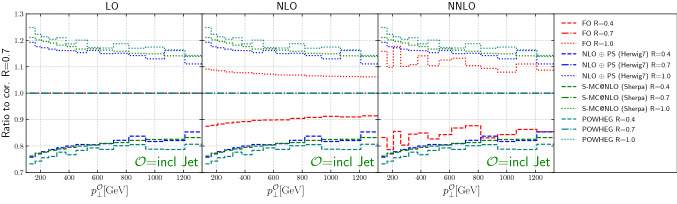
The ratio of the inclusive jet $p_T$ cross section for specific jet sizes, scaled to the cross section for each prediction for a jet size of $R=0.7$

Figure 13 shows the cross sections for the inclusive jet $p_T$ distribution for several different jet sizes, at LO, NLO and NNLO (from NNLOJET) and from the two NLO + PS predictions. The ratios to *R* = 0.7 decrease as a function of increasing jet $p_T$ at all orders. The differences between the three NLO+PS predictions and those from NNLOJET are of the order of 10% at NLO and of the order of 5% or less at NNLO.

Given the better description of the jet shape provided by the NLO+PS predictions, this is an indication of the theoretical uncertainty associated with the truncation of the perturbative series. The uncertainty is reduced at NNLO as expected. It is noteworthy that the ratios in Fig. 12 are relatively flat as a function of the transverse momenta.

**Fig. 14:**
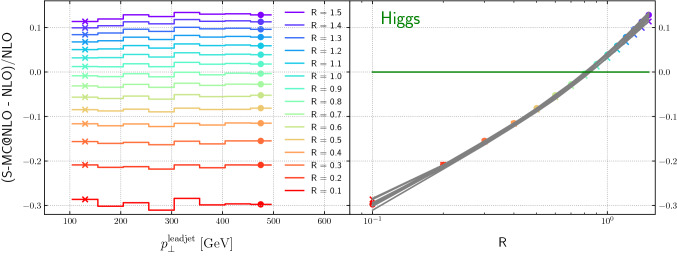
Relative difference between the NLO-matched prediction and the fixed-order result as a function of the leading jet transverse momentum and the jet radius, for $H+\ge 1$ jet

Figure 14 shows the dependence of the relative difference between a NLO-matched prediction from Sherpa and the NLO fixed-order result for $H+\ge 1$ jet production, as a function of the leading jet transverse momentum for varying jet radii. The ratio is flat as a function of the leading jet $p_T$. In Fig. 7 we compared integrated cross sections, while here we observe interestingly a similar behaviour for the differential cross sections. In the right plot, the projection is with respect to the radius, and displays, in grey, the various transverse momentum intervals and, in coloured, the lowest and highest energies. Assuming the leading behaviour is given by Eq. (7), and with the flatness in the leading jet transverse momentum, the linear, (but slightly quadratic) behaviour in the logarithmic plot is expected. We note the zero crossing of the curve on the right-hand side, which corresponds to the best agreement between fixed-order and NLO-matched result, is located at $R \approx$0.8 (see the discussion of Fig. 12). In configurations where the jet rapidity is zero, this corresponds to a roughly equal partitioning of the rapidity phase-space into collinear sectors for color dipoles spanned between the initial-state partons and the final-state jet, and thus to a roughly equal partitioning of soft-enhanced radiation. In the picture of angular ordered parton evolution, this corresponds to a natural separation of the phase space into the regions populated only by emissions from the closest jet. For smaller jet radii, radiation will leak from the jet, while for larger jet sizes radiation from other jets will be absorbed. We consider the beam parton as a jet of fixed axis. Due to the boost-invariance of the jet algorithm all final-state jets can be considered as central, $\eta =0$. This geometric argument favours the commonly used $R=0.7$ with respect to smaller values when experimental data is compared to fixed order calculations, although the precise value will depend on the color structure of the process and on the parton luminosity.

**Fig. 15:**
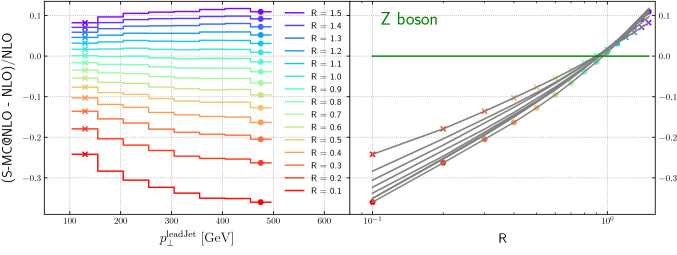
Relative difference between the NLO-matched prediction and the fixed-order result as a function of the leading jet transverse momentum and the jet radius, for $Z+\ge 1$ jet

Figure 15 shows the dependence of the relative difference between a NLO-matched prediction from Sherpa and the NLO fixed-order result, for $Z+\ge 1$ jet production, as a function of the leading jet transverse momentum for varying jet radii. In contrast to the Higgs boson case, the distributions are not flat as a function of lead jet transverse momentum for small-*R* jets and for large-*R* jets. Note also that the zero-crossing for the curves on the right-hand side is closer to $R=0.9$ than to $R=0.8$.

**Fig. 16:**
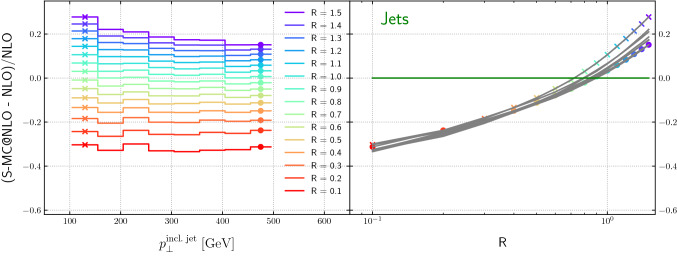
Relative difference between the NLO-matched prediction and the fixed-order result as a function of the inclusive jet transverse momentum and the jet radius, for dijet production

Figure 16 shows the dependence of the relative difference between a NLO-matched prediction from Sherpa and the NLO fixed-order result, for the inclusive jet transverse momentum, for dijet production, as a function of the inclusive jet transverse momentum for varying jet radii. The curves are relatively flat as a function of inclusive jet transverse momentum, for jet *R* values less than 0.7, but fall more steeply for larger *R* values. Note that the zero-crossings for the curves on the right-hand sides of the figures (for lead jet and inclusive jet) are around $R=0.8$.

**Fig. 17:**
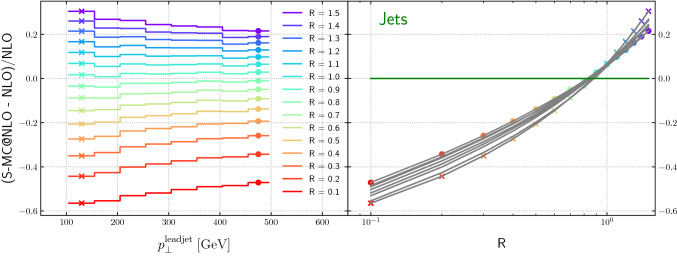
Relative difference between the NLO-matched prediction and the fixed-order result as a function of the leading jet transverse momentum and the jet radius, for dijet production

Figure 17 shows the dependence of the relative difference between a NLO-matched prediction from Sherpa and the NLO fixed-order result, for the lead jet transverse momentum, for dijet production, as a function of the leading jet transverse momentum for varying jet radii.^6^ The curves are relatively flat as a function of lead jet transverse momentum, for jet *R* values around 0.5, but fall (rise) more steeply for larger (smaller) *R* values.

Comparing the Figs. 14, 15, 16 and 17 we note the relative narrow distribution of grey lines that sample the different $p_T$ bins in the case of Higgs production. One might expect that this behavior is due to the Higgs production process being gluon-initiated. However, the decomposition into flavor channels shows that initial state quarks do play an important role and that quark-gluon initiated processes start to dominate for high transverse momenta. This diverse flavour composition of initial and final state does not allow us to make a definite statement without further studies.

**Fig. 18:**
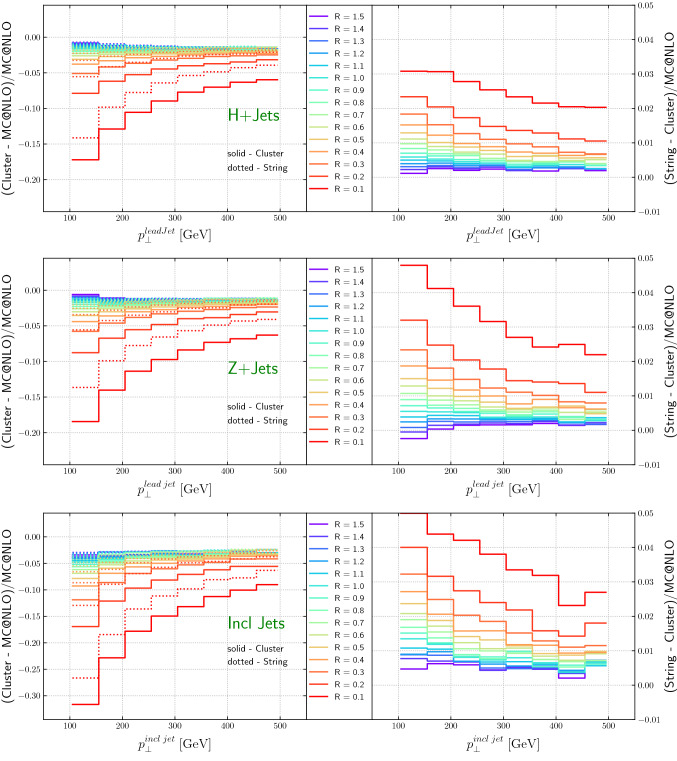
Hadronization corrections and uncertainties for Higgs + jets (top), Z + jets (middle) and inclusive jets (bottom)

**Fig. 19:**
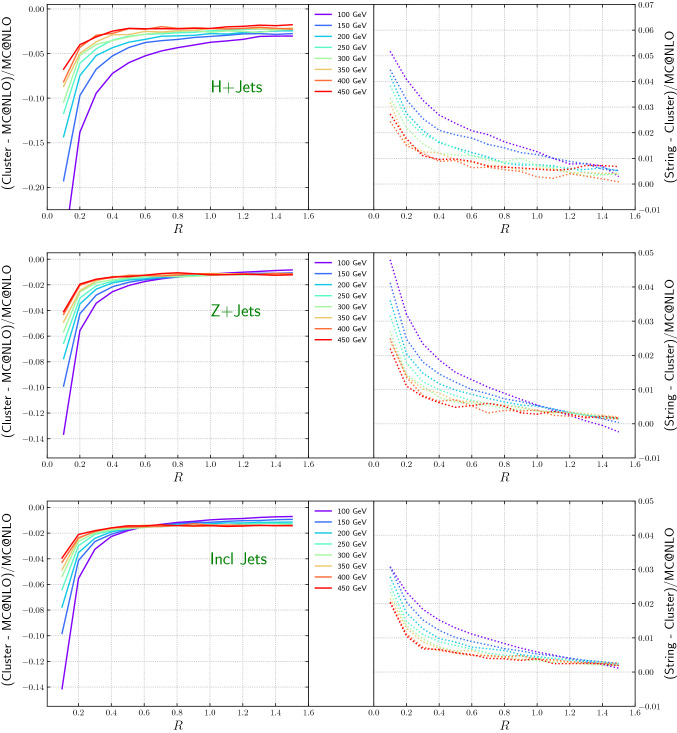
R-dependence of the hadronization corrections and uncertainties for Higgs + jets (top), Z + jets (middle) and inclusive jets (bottom)

**Fig. 20:**
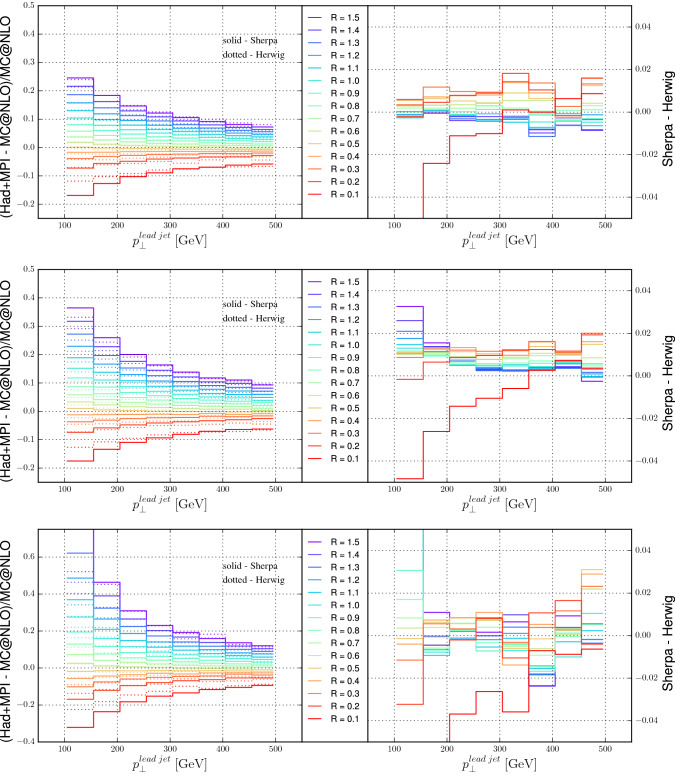
Hadronization plus MPI corrections for Higgs+jets (top), Z+jets (middle) and inclusive jets (bottom)

## Uncertainty estimates in processes with final-state jets

The reduction of scale uncertainties achievable at NNLO is remarkable. However, the *R*-dependence of the uncertainty discussed in Sect. 5 indicates that some of the improvements may be due to accidental cancellations. It is well known, that the scale variation for exclusive cross sections is prone to the accidental compensation of logarithmically enhanced higher-order corrections that appear both as a result of scale variations and as a result of the phase-space restrictions. The very definition of a final-state jet implies an exclusive measurement and effectively acts as a veto on real-radiative corrections that fall outside the jet area. This effect has been studied in different contexts [30, 111]. An accurate assessment of the perturbative uncertainties is important for inclusive jet production (and to a lesser extent for *Z* + jet production), as the PDF fitting groups are working to incorporate scale uncertainties in their analyses, and jet production serves as one of the major constraints on the gluon distribution, especially at large *x*. The impact is also especially important for smaller jet sizes (*R* = 0.4), commonly used for many measurements at the LHC, such as Higgs + jet production. The accidental cancellations can also be an issue at NLO, but it is less noticeable, given the larger intrinsic uncertainties at that order.

The *ansatz* advocated in [30] is to view the differential cross section as a combination of a fixed-order term and the normalized all-orders resummed result. The two are then combined through multiplicative matching, and their perturbative uncertainties are added in quadrature. Upon re-expanding this result to fixed-order, one obtains the $\mathrm {N}^n\mathrm {LO}$-mult prescription given in [30], Eqs. (3.5) and (4.3). The result can be written as

8 $$\begin{aligned}&\sigma (R)=\sigma (R_0)\frac{\sigma (R)}{\sigma (R_0)} \nonumber \\&\quad \approx \sigma (R_0)\cdot \left( 1+\alpha _S\, \partial _{\alpha _S} \frac{\sigma (R)}{\sigma (R_0)}\Big |_{\alpha _S=0}+\alpha ^2_S\, \partial ^2_{\alpha _S} \frac{\sigma (R)}{\sigma (R_0)}\Big |_{\alpha _S=0}\right) . \end{aligned}$$

Clearly there are several possible choices in regards to the implementation of the factorization of terms on the right-hand side of Eq. (8). Results from the original proposal in [30] are shown in the lower panels of Figs. 7, 8, 9, 10 and 11. We refer to this technique as “Ansatz 3”. While the red and blue dotted lines in Figs. 7, 8, 9, 10 and 11 correspond to typical scale variations the green dashed lines show the ratio of “Ansatz 3” (and other choices explained in the following) to the central scale prediction. The uncertainty of “Ansatz 3” has a more realistic-seeming value for all *R*, but the central value of the prediction is modified at small *R*, in some cases leading to the resultant uncertainty not encompassing the central value of the original NNLO prediction. We therefore investigate two alternative approaches. In the first (“Ansatz 1”), the ratio $\sigma (R)/\sigma (R_0)$ on the right-hand side of Eq. (8) is not expanded, and we combine the uncertainties from the ratio and the seed cross section $\sigma (R_0)$ in quadrature. The results of this procedure are shown in the top ratio panels of Figs. 7, 8, 9, 10 and 11. Our second alternative method (“Ansatz 2”), is based on the parametrization of the cross section as a function of *R* according to Eq. (7). We then determine the scale uncertainties of the fit coefficients *a*, *b* and *c* and combine them in quadrature to arrive at the full uncertainty. It can be seen in comparison between the top and middle ratio panels of Figs. 7, 8, 9, 10 and 11. that Ansatz 1 and Ansatz 2 give similar results, and both preserve the central value of the original NNLO fixed-order result. Although larger than the original uncertainties, the perturbative scale variations determined in this way are still smaller than the uncertainties observed at NLO, as would be expected from a higher order calculation (Fig. 18).

It is important to note that all of the aforementioned approaches of estimating the theoretical uncertainty from missing higher-order corrections have an intrinsic dependence on the arbitrary reference value $R_0$. By varying $R_0$ it is possible to create again a situation where the logarithmic corrections due to higher-order effects and due to phase-space restrictions compensate each other and the scale uncertainty is reduced to nearly zero. Based on the analysis in Sects. 3–5 we advocate to fix the reference radius $R_0$ by comparing the higher-order result to a parton-shower matched calculation and choose the reference point where the two (approximately) agree. As discussed in Sect. 5, this corresponds to selecting a reference radius where large logarithmic higher-order corrections are minimized. Here we choose $R_0=0.7$ for all uncertainty ansätze. Variations of $R_0$ in the ranges where the *R*-dependence can be well approximated by a linear fit around $R_0$, typically $R_0\in [0.5,1.0]$, have a mild impact on the uncertainty bands generated by the scale variations.

## Hadronization corrections and uncertainties

In this section we examine the non-perturbative corrections on the predictions presented before. We determine the hadronization uncertainties by taking the difference between NLO matched and hadronized results from Sherpa, using either the cluster fragmentation model as implemented in Sherpa [112] or an interface to the Lund string fragmentation model as implemented in Pythia [113].

Figure 18 (top and middle) shows that the hadronization corrections for the lead jet in $H+\ge 1$ jet and in $Z+\ge 1$ jet are very similar. For the commonly used jet size *R* = 0.4, the corrections are of the order of 5% or less. String fragmentation leads to slightly larger corrections, but the differences between the cluster and string fragmentation models are significantly smaller than the magnitudes of the corrections, on the order of 2% or less for $R=0.4$ and decreasing for larger *R*, as expected. For small jet radii, we observe the expected $1/p_T$ scaling of the hadronization corrections.

Figure 19 shows the cross-section as function of the jet radius for the various $p_T$-slices as well as the difference between string and cluster fragmentation. The expected 1/*R* dependence [114] is clearly visible. The change from cluster to string fragmentation reduces the effect slightly in the current setup but the general trend remains.

The pattern is similar for the inclusive jet transverse momentum spectrum for dijet production, as shown in Fig. 18 (bottom), although the impacts are magnified given the dijet final state at Born level. For *R* = 0.4, the difference between cluster and string fragmentation is of the order of 2.5% or less.

The combined corrections from hadronization and the underlying event, modeled through multiple parton interactions (MPI) are shown in Fig. 20. As the two corrections are in opposite directions, and are of similar magnitude for jet sizes of the order of 0.4, the combined correction is small, of the order of 2% or less for $R=0.4$, except for dijet production, where the combined correction can be as large as 5%. The related uncertainties shown on the right-hand side of Fig. 20 are determined by taking the difference between predictions from Sherpa and Herwig, both using their default MPI tunes and Cluster fragmentation.

## Conclusion and outlook

Searches for new physics, as well as a better understanding of standard model physics, require an increasing level of precision, both for measurement and for theory. For differential distributions, the highest level of precision is obtained with NNLO calculations. Matched NLO plus parton shower predictions (NLO+PS) start form less accurate fixed-order results, but provide a more complete description of the event structure, including resummation effects at leading logarithmic accuracy. Most physics measurements at the LHC make use of relatively small jet sizes (anti-$k_T$ with $R=0.4$), and $H(Z)+\ge 1$ jet production and dijet production are no exception. There can be differences between fixed order and NLO+PS predictions for the same observable just due to the different estimates of the amount of jet energy contained in a jet of radius *R*. These differences can be comparable to the size of the scale uncertainty for the cross section at that order.

In this contribution, we have reported on an investigation of the impact of different jet sizes on Higgs plus jet, *Z* boson plus jet, and dijet physics at the LHC, paying close attention to the impact of the jet size on *K*-factors, on scale uncertainties, and on differences between fixed order and NLO+PS predictions. Better understanding of the issues described here may allow an improvement in the accuracy, and precision, of such predictions at the LHC.

Our comparisons of the jet shapes for the three processes at fixed-order, full parton shower level, and truncated parton shower level indicated that the differences observed between fixed-order and NLO-matched results are due to higher parton multiplicity final states. We have observed the best agreement, for predictions involving jets, between fixed-order and NLO-matched predictions, occur when the jet size R is relatively large, and/or the fixed-order prediction is at NNLO compared to NLO. In the former, the jet shape is not as critical, and in the latter the jet shape is better described. We have found excellent agreement among the NLO-matched predictions for all observables.

The scale uncertainty naturally decreases in going from LO to NLO to NNLO, and also tends to decrease as the jet size decreases. We have observed that the (suitably normalized) NLO-matched results are within the fixed-order scale uncertainty bands at LO, NLO and NNLO, for $H+\ge 1$ jet for all jet sizes, but are typically outside the NNLO scale uncertainty bands for $Z+\ge 1$ jet, due to the very small values of the scale uncertainties for this process at this order. The scale uncertainties at NNLO can in fact be at or near zero for small jet sizes, indicating that the standard scale uncertainty paradigm does not provide an accurate description of the uncertainty of the calculation. These small uncertainties are due to accidental cancellations arising from the restriction of the phase space for small-R jets. We have constructed several ways of providing more robust determinations of the scale uncertainties.

Lastly, we have compared the non-perturbative predictions for all three processes as a function of jet size R and jet transverse momentum, and have found very good agreement between string and cluster fragmentation, and between the full non-perturbative corrections, fragmentation plus MPI, between Sherpa and Herwig.

In summary, we expect parton-shower matched predictions to differ from the underlying fixed-order results in regions where (1) there is a large sensitivity to jet shapes (typically small *R* jets), (2) there is another restriction in phase space such that soft gluon resummation effects become important, (3) the observable contains multiple, disparate scales, (4) the observable is sensitive to higher multiplicity final states than those described by the fixed-order calculation. Such differences should be smaller at NNLO than at NLO. Large parton shower effects in the absence of large higher-order corrections of type (1)–(4) should be viewed with suspicion, as should large differences between parton shower predictions in general.

## Data Availability

This manuscript has no associated data or the data will not be deposited. [Authors’ comment: This is a phenomenological study without producing experimental data. The results of the Monte Carlo simulations are presented in the figures.]

## Footnotes

1 Inclusive jet production has been measured at two different jet radii, for example *R* = 0.4 and 0.6 for ATLAS. The global PDF fiting groups almost always use the larger jet size, where the jet shape is not as critical.

2 As a reminder, the non-perturbative corrections used for fixed order predictions are determined from a comparison of the parton shower predictions with and without the non-perturbative effects, as a function of jet radius. This implicitly requires the integrated jet shape determined by fixed order predictions to agree with those determined by parton showers.

3 To make the analysis publically available, we add the used analysis files to the second arXiv version of this paper.

4 A similar comparision has been made for VBF and DIS in [107, 108] and recently discussing soft modifications in [109].

5 Note that this statement is dependent on the functional form used for the scale.

6 Note that for this distribution the *R*-dependence is larger than for any of the other distributions.
